# Syncope Due to a Ruptured Ectopic Pregnancy

**DOI:** 10.21980/J86M0N

**Published:** 2021-01-15

**Authors:** Derek JC Hunt, Kevin McLendon, Jodi Conrad

**Affiliations:** * Merit Health Wesley, Department of Emergency Medicine, Hattiesburg, MS

## Abstract

**Audience:**

This simulation is intended for all levels of emergency medicine residents.

**Introduction:**

Syncope and near-syncope are very common presenting complaints to the emergency department.^1^ There are several causes of syncope ranging from benign to life threatening. It is imperative that the emergency physician is able to evaluate and treat patients with undifferentiated syncope even with limited information. Approximately half of syncope cases can be differentiated by the presentation and clinical context.^1^ In addition to a thorough history, an electrocardiogram (ECG) should be obtained on all patients presenting with syncope or near-syncope since it is non-invasive and cost effective in assessing cardiac causes of syncope. In this particular simulation, the cause of syncope is due to a hemorrhagic shock from a ruptured ectopic pregnancy.

**Educational Objectives:**

At the conclusion of this simulation, the learner will be able to:

1. Review the initial management of syncope
2. Utilize laboratory and imaging techniques to diagnose a ruptured ectopic pregnancy.
3. Demonstrate the ability to resuscitate and disposition an unstable ruptured ectopic pregnancy.

**Educational Methods:**

This simulation case was designed as a medium-to-high fidelity simulation scenario. It could also be altered and utilized as a practice oral board exam case.

**Research Methods:**

The quality of the simulation and educational content was evaluated by debriefing and verbal feedback that was obtained immediately after the case. Additionally, a survey was emailed to participants and observers of the case to provide qualitative feedback.

**Results:**

Post-simulation feedback from learners and observers was positive. Everyone present for the simulation felt that it was realistic and provided a unique opportunity to practice resuscitation skills.

**Discussion:**

Syncope and near-syncope are common presentations to the emergency department with multiple etiologies that range from cardiac, neurologic, neurocardiogenic, and orthostatic to unknown. It is crucial that we diagnose and treat life-threatening causes of syncope rapidly and with limited information. In this case, the cause of syncope due to a ruptured ectopic pregnancy should be rapidly diagnosed with a thorough history and exam, urine pregnancy test, and a bedside abdominal ultrasound. Once the urine pregnancy test was resulted, ectopic pregnancy was the top differential diagnosis for each learner that participated. Initially, most learners only performed a transabdominal pelvic ultrasound of the pelvis, which is normal in the case. One learner performed a rapid ultrasound for shock and hypotension (RUSH) exam and was able to find free fluid in the right upper quadrant. Overall, this case and the debriefing topics were effective for learners at all levels.

**Topics:**

Ectopic pregnancy, obstetrical emergencies, ultrasound, resuscitation.

## USER GUIDE

[Table t1-jetem-7-1-s1]

**Table t1-jetem-7-1-s1:** 

List of Resources:	
Abstract	1
User Guide	3
Instructor Materials	5
Operator Materials	12
Debriefing and Evaluation Pearls	14
Simulation Assessment	16

**Learner Audience:**

Emergency Medicine residents

**Time Required for Implementation:**

Instructor Preparation: 30 minutes

Time for case: 15–20 minutes

Time for debriefing: 10–15 minutes

**Recommended Number of Learners per Instructor:**

1–2

**Topics:**

Ectopic pregnancy, obstetrical emergencies, ultrasound, resuscitation.

**Objectives:**

At the conclusion of this simulation, the learner will be able to:

1. Review the initial management of syncope.
2. Utilize laboratory and imaging techniques to diagnose a ruptured ectopic pregnancy.
3. Demonstrate the ability to resuscitate and disposition an unstable ruptured ectopic pregnancy.

### Linked objectives and methods

Syncope and near-syncope are common presenting symptoms to the emergency department with a very broad range of differential diagnoses. The presentation and circumstances around the syncopal event should help guide emergency physicians on how to evaluate and determine the cause of syncope (objective 1). At a minimum, an electrocardiogram should be ordered on every patient presenting to the emergency department with syncope or near-syncope (objective 1). In patients of childbearing age, obstetrical emergencies should be considered, particularly hemorrhagic shock due to a ruptured ectopic pregnancy, which can cause syncope, and it is important for emergency physicians to rapidly diagnose and treat it. A urine pregnancy test and ultrasound can be done rapidly and at the bedside when evaluating a potentially ruptured ectopic pregnancy (objective 2). Once the patient has been correctly diagnosed with hemorrhagic shock from an ectopic pregnancy, the learner should begin to resuscitate the patient and order an emergency release of packed red blood cells and obtain an emergent OBGYN consult (objective 3).

### Learner responsible content

- Heaton H. Ectopic Pregnancy and Emergencies in the First 20 Weeks of Pregnancy. In: *Tintinalli’s Emergency Medicine: A Comprehensive Study Guide* (9th ed.). United States: McGraw-Hill Medical Publishing Division; 2020: 615–622.
- Quinn J. Syncope. In: *Tintinalli’s Emergency Medicine: A Comprehensive Study Guide.* 9th ed. United States: McGraw-Hill Medical Publishing Division; 2020:362–367.

### Recommended pre-reading for instructor

- Heaton H. Ectopic Pregnancy and Emergencies in the First 20 Weeks of Pregnancy. In: *Tintinalli’s Emergency Medicine: A Comprehensive Study Guide* (9th ed.). United States: McGraw-Hill Medical Publishing Division; 2020: 615–622.
- Quinn J. Syncope. In: *Tintinalli’s Emergency Medicine: A Comprehensive Study Guide.* 9th ed. United States: McGraw-Hill Medical Publishing Division; 2020:362–367.

### Results and tips for successful implementation

The simulation was performed during our weekly didactic sessions as one of three small group activities. Two learners participated during each session with approximately 4–5 observers which consisted of emergency medicine residents and medical students. Learners were evaluated by the instructor(s) based on their ability to correctly diagnose and treat the patient. Initial impressions from the participants and observers were obtained verbally during the debriefing session which occurred immediately after the case. A survey was also emailed to all participants and observers to assess the quality of the case and is available in the pie graphs below. Participant feedback was overall very positive, and they did not recommend any significant changes to the case design or format. There were no modifications to the case after the initial implementation.[Fig f1-jetem-7-1-s1]

**Figure f1-jetem-7-1-s1:**
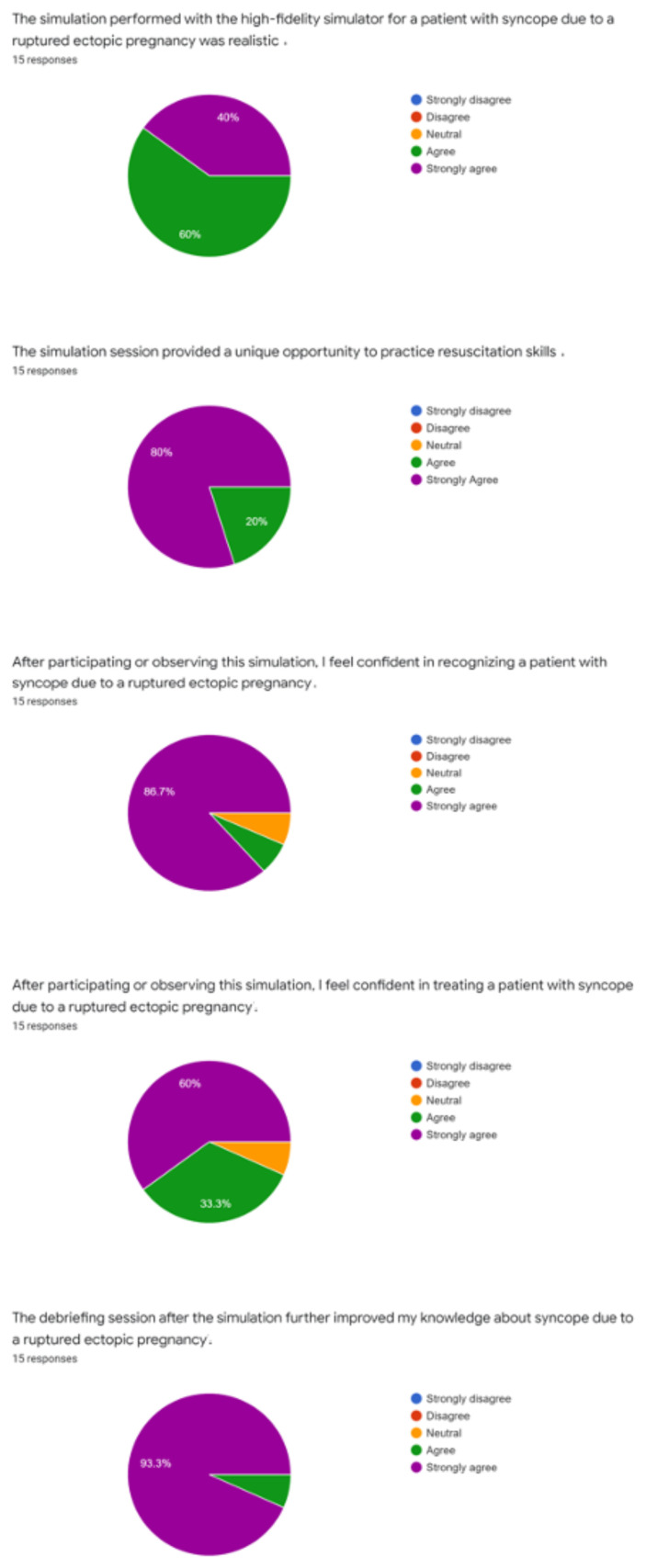


## Supplementary Information



## Figures and Tables

**Table t2-jetem-7-1-s1:** 

#1	Urine or serum hCG
#2	Right Upper Quadrant ultrasound from FAST exam (Focused Assessment with Sonography for Trauma) with free fluid in Morrison’s pouch
#3	Electrocardiogram

**Table t3-jetem-7-1-s1:** 

**Vitals:**	Heart Rate (HR) 115	Blood Pressure (BP) 80/540	Respiratory Rate (RR) 22
	Temperature (T) 98.5°F	Oxygen Saturation (O_2_Sat) 99% on room air	

**Table t4-jetem-7-1-s1:** 

*Qualitative hCG Urine:*	Positive
*Qualitative hCG Serum:*	Positive (if used)

**Figure f2-jetem-7-1-s1:**
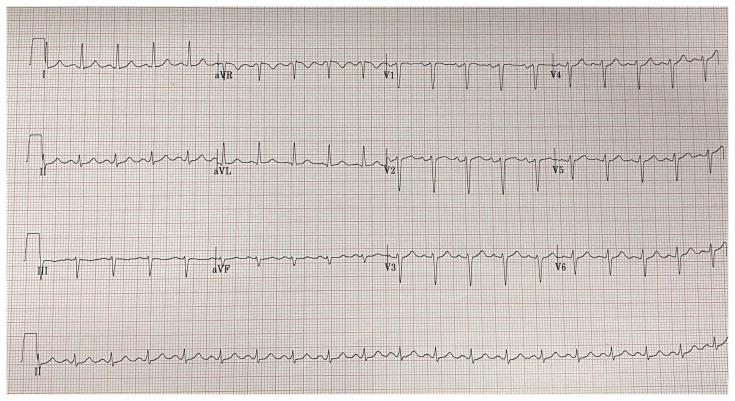


**Figure f3-jetem-7-1-s1:**
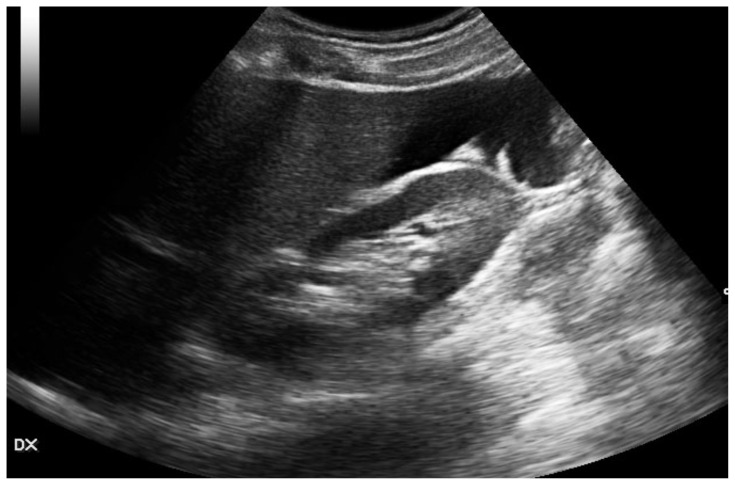


**Table t5-jetem-7-1-s1:** SIMULATION EVENTS TABLE:

Minute (state)	Participant action/trigger	Patient status (simulator response) & operator prompts	Monitor display (vital signs)
0:00 State 1	Patient placed on cardiac monitor Requests 2 large bore IVs Vitals obtained Gather HPI Perform primary and secondary survey Request ECG and urine hCG	Nurse requests assistance and husband present to provide history. Patient presents with RLQ pain and has a syncopal episode while walking to triage. She is lying supine in bed, pale, and mildly ill appearing. Learner(s) may order an IV fluid bolus, but the vital signs will only transiently improve with the first liter. Additional fluid boluses will not have an effect on vital signs or clinical condition. If the learner attempts orthostatic vitals, the patient will have another syncopal episode.	T 98.5 HR 115 BP 80/40 RR 22 O2 99% RA
State 2 (Begins after history and exam completed and nurse notifies learner of positive urine hCG)	Performs bedside US since formal US unavailable for 2 hours Orders CBC, BMP/CMP, and type and screen Requests emergent release of O-negative blood May consider activating massive transfusion protocol (MTP) to be on stand-by	Nurse notifies learner of positive urine pregnancy test. If emergent release of O-negative is requested, transition to State 4 (blood received) after a few minutes. If incorrect type of blood or no blood is requested, transition to State 3 (asystole). If O-negative blood is not requested within 10 minutes, transition to State 3 (asystole). If learner requests a CT abdomen/pelvis, the patient will code while on the CT table, transition to State 3 (asystole).	T 98.5 HR 123 BP 75/40 RR 21 O2 99% RA
State 3 (asystole)	Performs 3 rounds of CPR Performs intubation Requests emergent release of O-negative blood	This state only occurs if the learner(s) order a CT or plan to wait for a formal US to be performed. Patient will obtain ROSC after 3 rounds of CPR and endotracheal intubation. O-negative blood should be requested during CPR or after ROSC. If not requested within 2 minutes of ROSC, the patient will return to asystole, ROSC will not be obtained, and the patient will expire.	T 98.5 HR 0 BP 0/0 RR 0 O2 99% bag valve mask, endotracheal intubation
State 4 (blood received and case completion)	Consults OBGYN Requests additional blood products when hypotension returns Activates massive transfusion protocol (MTP) once 4 units of packed red blood cells are ordered (Optional) Inserts cordis catheter or equivalent large bore access	Patient reports feeling better after receiving initial blood products. OBGYN and operating room will be ready in 30 minutes. While waiting for surgery, the patient becomes more hypotensive. This will improve when additional blood products are given and MTP is activated.	T 98.5 HR 102 BP 90/70 RR 21 O2 99% RA If assessing activation of MTP: T 98.5 HR 135 BP 70/30 RR 21 O2 99% RA

**Table t6-jetem-7-1-s1:** 

**Critical Actions:** Order an electrocardiogram and interpret as sinus tachycardia Order urine hCG (serum QUALitative may be used but QUANTitative will not result in timely fashion) Perform bedside ultrasound and recognize free fluid in Morrison’s pouch Obtain emergent release of O-negative blood Order type and screen Consult OBGYN Activate or prepare blood bank for massive transfusion protocol	0:00

**Table t7-jetem-7-1-s1:** 

	Milestone	Did not achieve level 1	Level 1	Level 2	Level 3
1	**Emergency Stabilization (PC1)**	□ Did not achieve Level 1	□ Recognizes abnormal vital signs	□ Recognizes an unstable patient, requiring intervention Performs primary assessment Discerns data to formulate a diagnostic impression/plan	□ Manages and prioritizes critical actions in a critically ill patient Reassesses after implementing a stabilizing intervention
2	**Performance of focused history and physical (PC2)**	□ Did not achieve Level 1	□ Performs a reliable, comprehensive history and physical exam	□ Performs and communicates a focused history and physical exam based on chief complaint and urgent issues	□ Prioritizes essential components of history and physical exam given dynamic circumstances
3	**Diagnostic studies (PC3)**	□ Did not achieve Level 1	□ Determines the necessity of diagnostic studies	□ Orders appropriate diagnostic studies. Performs appropriate bedside diagnostic studies/procedures	□ Prioritizes essential testing Interprets results of diagnostic studies Reviews risks, benefits, contraindications, and alternatives to a diagnostic study or procedure
4	**Diagnosis (PC4)**	□ Did not achieve Level 1	□ Considers a list of potential diagnoses	□ Considers an appropriate list of potential diagnosis May or may not make correct diagnosis	□ Makes the appropriate diagnosis Considers other potential diagnoses, avoiding premature closure
5	**Pharmacotherapy (PC5)**	□ Did not achieve Level 1	□ Asks patient for drug allergies	□ Selects an medication for therapeutic intervention, consider potential adverse effects	□ Selects the most appropriate medication and understands mechanism of action, effect, and potential side effects Considers and recognizes drug-drug interactions
6	**Observation and reassessment (PC6)**	□ Did not achieve Level 1	□ Reevaluates patient at least one time during case	□ Reevaluates patient after most therapeutic interventions	□ Consistently evaluates the effectiveness of therapies at appropriate intervals
7	**Disposition (PC7)**	□ Did not achieve Level 1	□ Appropriately selects whether to admit or discharge the patient	□ Appropriately selects whether to admit or discharge Involves the expertise of some of the appropriate specialists	□ Educates the patient appropriately about their disposition Assigns patient to an appropriate level of care (ICU/Tele/Floor) Involves expertise of all appropriate specialists
9	**General Approach to Procedures (PC9)**	□ Did not achieve Level 1	□ Identifies pertinent anatomy and physiology for a procedure Uses appropriate Universal Precautions	□ Obtains informed consent Knows indications, contraindications, anatomic landmarks, equipment, anesthetic and procedural technique, and potential complications for common ED procedures	□ Determines a back-up strategy if initial attempts are unsuccessful Correctly interprets results of diagnostic procedure
20	**Professional Values (PROF1)**	□ Did not achieve Level 1	□ Demonstrates caring, honest behavior	□ Exhibits compassion, respect, sensitivity and responsiveness	□ Develops alternative care plans when patients’ personal beliefs and decisions preclude standard care
22	**Patient centered communication (ICS1)**	□ Did not achieve level 1	□ Establishes rapport and demonstrates empathy to patient (and family) Listens effectively	□ Elicits patient’s reason for seeking health care	□ Manages patient expectations in a manner that minimizes potential for stress, conflict, and misunderstanding. Effectively communicates with vulnerable populations, (at risk patients and families)
23	**Team management (ICS2)**	□ Did not achieve level 1	□ Recognizes other members of the patient care team during case (nurse, techs)	□ Communicates pertinent information to other healthcare colleagues	□ Communicates a clear, succinct, and appropriate handoff with specialists and other colleagues Communicates effectively with ancillary staff

## INSTRUCTOR MATERIALS

**Case Title:** Syncope Due to a Ruptured Ectopic Pregnancy

**Case Description & Diagnosis (short synopsis):** 24-year-old female presents to the emergency department for worsening right lower quadrant abdominal pain with nausea and vomiting. When she is called from the waiting room by the triage nurse, she has a syncopal episode and is brought back to a room immediately. A confederate who acts as the nurse calls for the learner to assess the patient. History is provided by another confederate who acts as the patient’s husband. The patient will ultimately be found to have a ruptured ectopic pregnancy diagnosed by bedside ultrasound. The patient will require an emergent blood transfusion and emergent OBGYN consult for surgical removal of the ectopic pregnancy.

**Equipment or Props needed:**

Medium or High-fidelity simulator

Cardiac monitor

Ultrasound machine

Electrocardiogram with sinus tachycardia

**Optional Equipment or Props needed:**

IV poles

Fluids/Fake Blood

Central Line task trainer

Intubating equipment

Cordis catheter

**Confederates needed:**

Nurse

Patient’s husband

OB/GYN consultant

**Stimulus Inventory:** [Table t2-jetem-7-1-s1]

**Background and brief information:** Patient presents to a community emergency department via private vehicle from home with her husband due to right lower quadrant abdominal pain with nausea and vomiting beginning that morning.

**Initial presentation:** Patient presents due to right lower quadrant abdominal pain with nausea and vomiting. When called from the waiting room to triage, she has a syncopal event and is brought back to a room immediately where she is found to be hypotensive.

**How the scene unfolds:** After learners perform their initial assessment, they should request an electrocardiogram (ECG) and a urine hCG. If serum hCG requested, only a qualitative will result, and the quantitative will be pending for the duration of the case. The ECG will be sinus tachycardia, and she will have unstable vital signs. Once the history has been gathered and the physical exam completed, or at the instructor’s discretion, the learners will be notified by the confederate nurse of the positive pregnancy test. A formal transabdominal and transvaginal ultrasound will not be available to be performed for two hours; therefore, the learners must perform their own ultrasound which will reveal free fluid in the right upper quadrant (Morrison’s Pouch) and no intrauterine pregnancy. The learners should request an emergent release of two units of O-negative blood. They may start with maximum 1L crystalloid; however, without blood the patient will deteriorate. The patient’s condition will improve, and then OBGYN should be consulted for surgical repair of an ectopic pregnancy. While waiting for transport to the operating room, the patient will again become hypotensive. The learners should recognize that the patient requires additional packed red blood cells and activation of the massive transfusion protocol.

If the learners do not request emergent release of O-negative blood, or if the learners do not recognize that the patient is having an ectopic pregnancy, she will go into asystole and require three minutes of cardiopulmonary resuscitation (CPR) and endotracheal intubation before she obtains return of spontaneous circulation (ROSC).

**Critical actions:**

1. Order an electrocardiogram and interpret as sinus tachycardia
2. Order urine hCG (serum QUALitative may be used but QUANTitative will not result in timely fashion)
3. Perform bedside ultrasound and recognize free fluid in Morrison’s pouch
4. Obtain emergent release of O-negative blood
5. Order type and screen
6. Consult OBGYN
7. Activate or prepare blood bank for massive transfusion protocol

**Case Title:** Syncope Due to a Ruptured Ectopic Pregnancy

**Chief Complaint:** Lower abdominal pain with nausea and vomiting, but patient has a syncopal episode while walking to triage[Table t3-jetem-7-1-s1]

**General Appearance:** alert, pale, mildly ill-appearing

**Primary Survey:**

- **Airway:** speaks in full sentences, airway patent
- **Breathing:** mildly tachypneic, clear and equal breath sounds
- **Circulation:** hypotensive, tachycardic, peripheral pulses weak but equal bilaterally

**History:**

- **History of present illness:** (History provided by husband/confederate) 24-year-old female presenting with right lower quadrant abdominal pain with nausea and vomiting. She has a syncopal episode while walking to the triage room and is instead brought back to a room immediately. The pain began that morning and steadily worsened throughout the day until it reached its peak intensity at home just prior to arrival. Nothing made the pain better or worse. Just before she passed out, she reported feeling very weak. She denies headache, CP, shortness of breath, vaginal bleeding, vaginal discharge, or urinary symptoms. She denies taking any medications for her symptoms. Her last menstrual cycle ended approximately 6 weeks ago, but her cycles are typically irregular.
- **Past medical history:** None
- **Past surgical history:** None
- **Medications:** Multivitamin
- **Allergies:** None
- **Social history:** Occasional alcohol, no tobacco, no illicit drugs
- **Family history:** Father with hypertension

**Secondary Survey/Physical Examination:**

- **General appearance:** alert, pallor, mildly ill-appearing
- **HEENT:**
  ○ **Head:** Normal
  ○ **Eyes:** Normal
  ○ **Ears:** Normal
  ○ **Nose:** Normal
  ○ **Throat:** Normal
- **Neck:** No jugular vein distention, no midline tenderness, trachea midline
- **Heart:** Tachycardic with regular rhythm, no murmurs, rubs, or gallops, peripheral pulses equal bilaterally
- **Lungs:** No respiratory distress, clear to auscultation bilaterally, symmetrical chest rise
- **Abdominal/GI:** Moderate to severe tenderness in the lower abdomen with involuntary guarding (must ask about guarding), positive rebound tenderness in the RLQ and suprapubic regions (must ask about rebound tenderness), no distention, no bruising
- **Genitourinary:** If the learner performs a pelvic exam; mild amount of blood in the vaginal vault, cervical os open
- **Extremities:** No edema, 5/5 muscles strength in the bilateral upper and lower extremities
- **Back:** Nontender, no step-offs
- **Neuro:** Alert and oriented x 4, no focal neurological deficits, answers all questions appropriately
- **Skin:** Pallor, no abrasions or lacerations, no rash
- **Lymph:** No lymphadenopathy
- **Psych:** Normal mood and affect

**Results:**

The learner is expected to order labs; however none of them will be resulted during the simulation other than the following:[Table t4-jetem-7-1-s1]

*Electrocardiogram:*

(author’s own image)[Fig f2-jetem-7-1-s1]

*RUQ component of the FAST/RUSH Exam demonstrating free fluid in Morrison’s pouch*

Image source: Adamoli P, 2022. *Hemoperitoneum | Radiology Case | Radiopaedia.org*. [online] Radiopaedia.org. Available at: <https://radiopaedia.org/cases/haemoperitoneum> [Accessed 6 January 2022].[Fig f3-jetem-7-1-s1]

## OPERATOR MATERIALS

[Table t5-jetem-7-1-s1]

**Diagnosis:**

Ruptured ectopic pregnancy

**Disposition:**

Operating room with OBGYN

## DEBRIEFING AND EVALUATION PEARLS

### Syncope Due to a Ruptured Ectopic Pregnancy

**Reaction phase questions:**

1. How do you feel you managed this case and what are your initial reactions?
2. What went well during the case and why?
3. Is there anything you would have done differently?
4. Were there any aspects of the case that made it more difficult and how did you manage these?
5. How did the patient’s syncopal event in triage make the initial assessment more difficult?

**Understanding phase questions:**

1. When a patient presents with syncope, what are other life-threatening causes to be considered? *Ie, dysrhythmia, hypoglycemia, seizure, pulmonary embolism, acute myocardial infarction, etc.*
2. If you could order one test/lab/imaging modality in a patient presenting with syncope, what would it be (ask each person present during the simulation)? *(FAST exam, RUSH exam, CXR, EKG, CMP, etc.)*
3. How would you manage this patient if a urine sample was not readily available? (*POCUS*, *serum hCG, etc.)*
4. How would a negative pregnancy test in this same patient change your management and decision making? (*FAST exam and gen surgery consult; look for additional history clues like trauma, etc; consider shock from other sources such as septic or cardiogenic)*

**Pearls:**

1. All patients, regardless of age, who present with syncope or near-syncope, should have an electrocardiogram performed. The remainder of their work-up is determined by features of their history, physical exam, and ECG results.
2. The remainder of a workup should be dictated by the history and physical; remember that undifferentiated syncope is high risk, especially in cases without a prodrome.
3. Always assess for pregnancy in women of childbearing age.
  - Loss of consciousness/syncope/near-syncope/abdominal pain in pregnancy is ectopic until proved otherwise.
4. When assessing for free fluid during a bedside ultrasound in a ruptured ectopic pregnancy, perform a FAST/RUSH exam because fluid can be seen in areas other than the pelvis.
5. Crystalloids may be used for initial resuscitation of hypotensive patients, but in hypovolemic shock due to a ruptured ectopic pregnancy, blood should be used early, just as in trauma resuscitation.
6. Massive transfusion protocols may be different in every hospital, but activation should typically occur when 4–6 units of packed red blood cells have been ordered.
7. Consultation with OBGYN should occur early in a hypotensive pregnant patient with abdominal pain. If OBGYN is not available at your facility, general surgery should be consulted. Without surgical intervention, you cannot treat the underlying cause.

## SIMULATION ASSESSMENT

*Syncope Due to a Ruptured Ectopic Pregnancy*

Learner: _________________________________________

***Assessment Timeline***

This timeline is to help observers assess their learners. It allows observer to make notes on when learners performed various tasks, which can help guide debriefing discussion.[Table t6-jetem-7-1-s1]

**Critical Actions:**

□ Order an electrocardiogram and interpret as sinus tachycardia
□ Order urine hCG (serum QUALitative may be used but QUANTitative will not result in timely fashion)
□ Perform bedside ultrasound and recognize free fluid in Morrison’s pouch
□ Obtain emergent release of O-negative blood
□ Order type and screen
□ Consult OBGYN
□ Activate or prepare blood bank for massive transfusion protocol

**Summative and formative comments:**

**Milestones assessment:** [Table t7-jetem-7-1-s1]
