# Supplementary material for: Syncope Due to a Ruptured Ectopic Pregnancy
Source: J Educ Teach Emerg Med. 2021 Jan 15;7(1):S1–S20. doi: 10.21980/J86M0N (PMC10358872; doi:10.21980/J86M0N)
Supplement: Supplementary file 1 [file JETem-7-1-S1-supp1.pptx]

## Slide 1
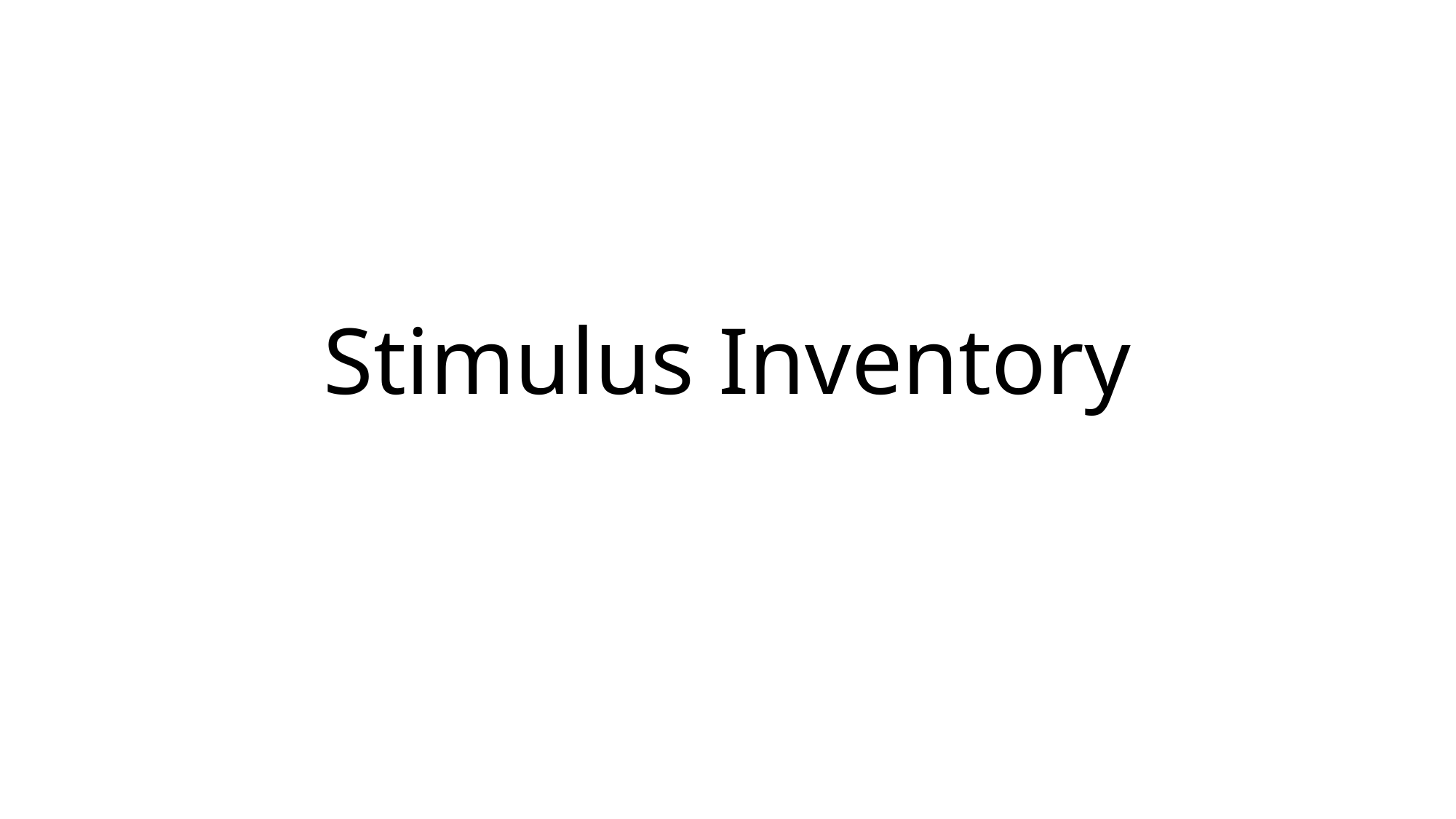

# Stimulus Inventory

## Slide 2
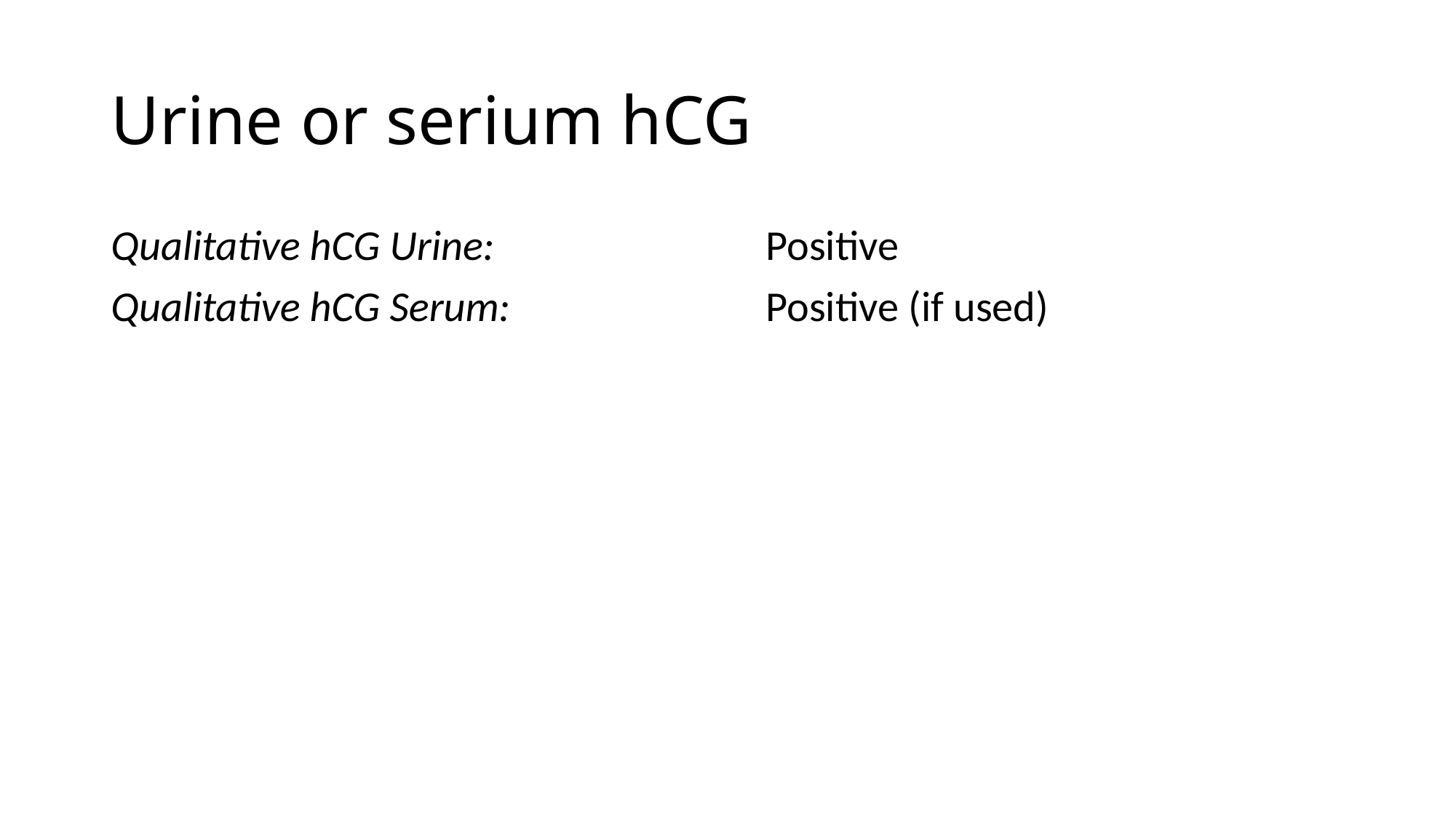

# Urine or serium hCG
Qualitative hCG Urine: 			Positive
Qualitative hCG Serum: 			Positive (if used)

## Slide 3
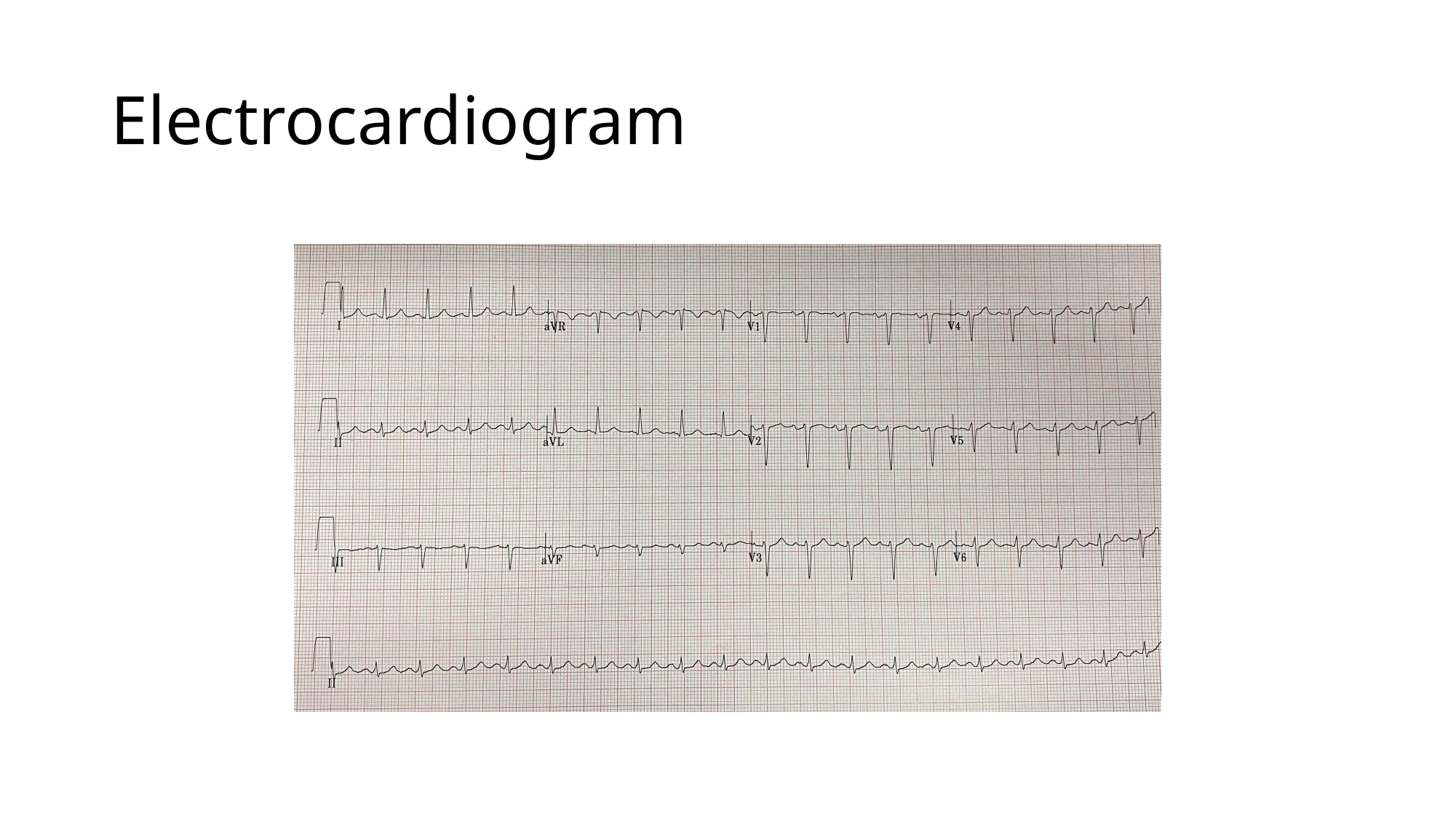

# Electrocardiogram

## Slide 4
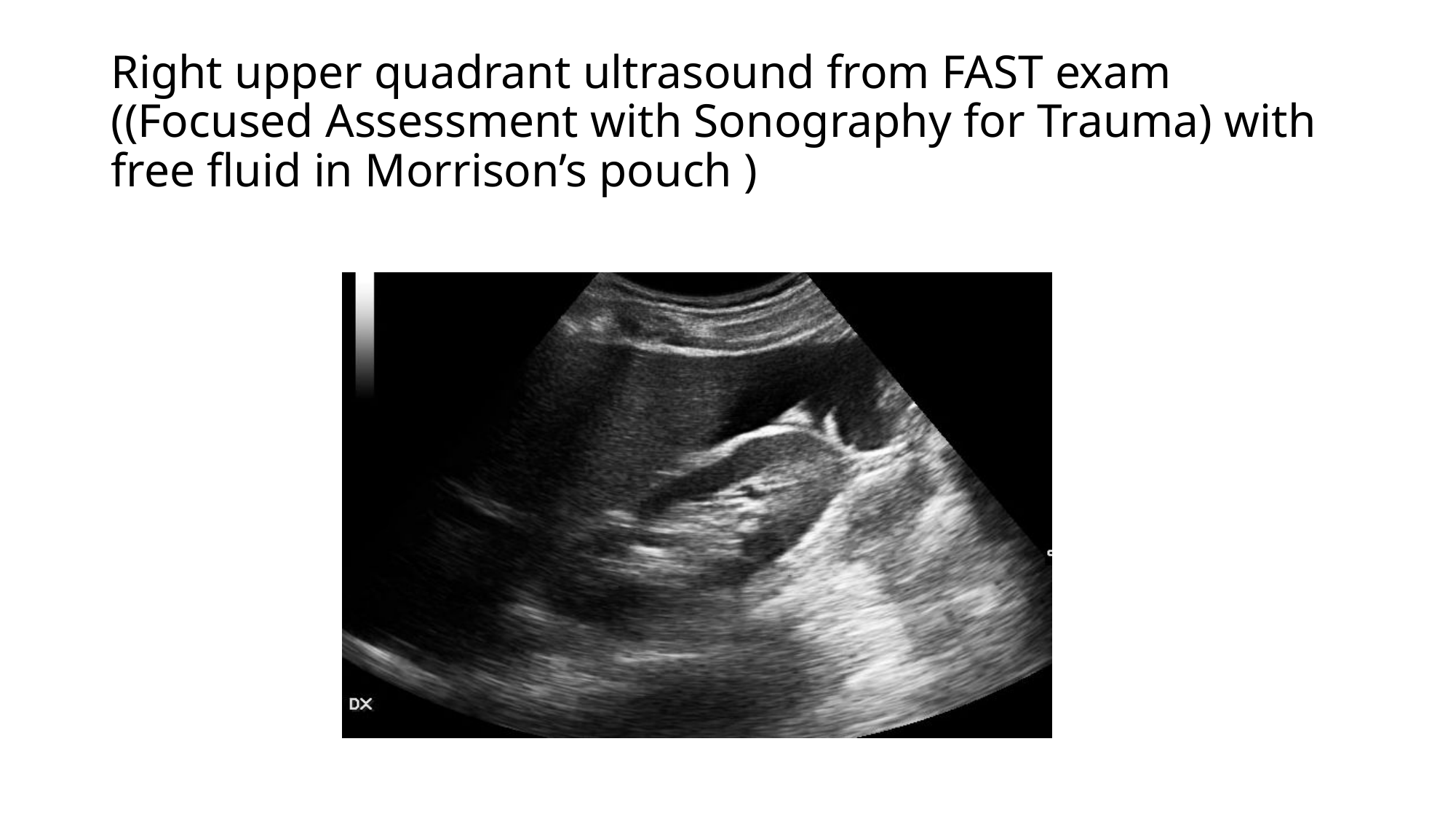

# Right upper quadrant ultrasound from FAST exam ((Focused Assessment with Sonography for Trauma) with free fluid in Morrison’s pouch )
